# Is the APLS formula used to calculate weight-for-age applicable to a Trinidadian population?

**DOI:** 10.1186/1471-227X-12-9

**Published:** 2012-08-02

**Authors:** Khalid Ali, Ian Sammy, Paula Nunes

**Affiliations:** 1 Eric Williams Medical Sciences Complex, Champs Fleurs, Trinidad, West Indies; 2 Faculty of Medical Sciences, The University of the West Indies, St Augustine, Trinidad, West Indies

## Abstract

**Background:**

In paediatric emergency medicine, estimation of weight in ill children can be performed in a variety of ways. Calculation using the ‘APLS’ formula (weight = [age + 4] × 2) is one very common method. Studies on its validity in developed countries suggest that it tends to under-estimate the weight of children, potentially leading to errors in drug and fluid administration. The formula is not validated in Trinidad and Tobago, where it is routinely used to calculate weight in paediatric resuscitation.

**Methods:**

Over a six-week period in January 2009, all children one to five years old presenting to the Emergency Department were weighed. Their measured weights were compared to their estimated weights as calculated using the APLS formula, the Luscombe and Owens formula and a “best fit” formula derived (then simplified) from linear regression analysis of the measured weights.

**Results:**

The APLS formula underestimated weight in all age groups with a mean difference of −1.4 kg (95% limits of agreement 5.0 to −7.8). The Luscombe and Owens formula was more accurate in predicting weight than the APLS formula, with a mean difference of −0.4 kg (95% limits of agreement 6.9 to −6.1%). Using linear regression analysis, and simplifying the derived equation, the best formula to describe weight and age was (weight = [2.5 x age] + 8). The percentage of children whose actual weight fell within 10% of the calculated weights using any of the three formulae was not significantly different.

**Conclusions:**

The APLS formula slightly underestimates the weights of children in Trinidad, although this is less than in similar studies in developed countries. Both the Luscombe and Owens formula and the formula derived from the results of this study give a better estimate of the measured weight of children in Trinidad. However, the accuracy and precision of all three formulae were not significantly different from each other. It is recommended that the APLS formula should continue to be used to estimate the weight of children in resuscitation situations in Trinidad, as it is well known, easy to calculate and widely taught in this setting.

## Background

In paediatric emergencies, drug dosages and fluids are administered according to weight [1]. In many cases it is impractical to weigh seriously ill children; it then becomes necessary to estimate the weight. At the Paediatric Emergency Department, Eric Williams Medical Sciences Complex, the Advanced Paediatric Life Support (APLS) formula is used to estimate weight in children from one to ten years of age. The original formula is as follows: Weight (kg) = (Age (years) + 4) × 2 [2].

Recently, much concern has been raised about the applicability of the APLS formula to modern day children, with several studies finding that the APLS formula tends to underestimate weight. Other methods that have been used to estimate weight in children are the Best Guess method, parental estimate, doctor’s estimate, nurse’s estimate, the Broselow tape and the Argall formula [3-9]. Many of these methods have produced better estimations of weight when compared to the APLS formula [3-7]. In particular, Luscome and Owens devised a formula from the weights of over 17 000 children in Sheffield, which appears to better estimate weight of children in developed countries. This formula (weight = (3 × age) +7) was shown to be more accurate for estimating weight, especially in the 6 – 12 years age group [3].

In light of the above, the most recent edition of the APLS manual describes three separate formulae for the estimation of weight in children: the original formula for children between the ages of 1 – 5, the Luscombe and Owens formula for those aged 6 – 12 and a specific infant formula for those aged less than 1 year old [2].

A search of the literature performed on Pubmed® on March 2^nd^ 2008 by the authors did not reveal any published literature on the applicability of the APLS formula to a Trinidadian or Caribbean population.

This study aimed to determine whether the APLS formula is applicable to children in Trinidad, specifically children aged 1 to 5 years. Our primary question is: does the APLS formula more accurately estimate weight than the Luscombe and Owens formula in children aged 1 to 5 years in Trinidad? The secondary question is: is there a more accurate formula than either the APLS or the Luscombe and Owens that can be used to estimate weight in this age group?

## Methods

This was an observational study of children presenting to the Emergency Department at the Eric Williams Medical Sciences Complex (EWMSC) over a six-week period from January 1^st^ to February 12^th^ 2009. This hospital is the only dedicated paediatric hospital in Trinidad and Tobago. All patients aged 1 to 5 years of age who attended the Paediatric Emergency Department were included in the study. The exclusion criteria were patients in whom age or weight were not performed, not documented, or not clearly documented. Patients older than 5 years on admission were also excluded from the study. Clinical records were reviewed retrospectively on patients who met the inclusion criteria. The research study was granted approval by The Eric Williams Medical Sciences Complex Ethics Committee.

Data collection included details of age at last birthday and weight. The Seca infant scale or Detecto standing scale was used by the triage nurse to measure weights. For uncooperative children, a subtraction method was used. The child’s weight was the difference between the combined weight of the parent and child and the weight of the parent alone. Both scales were calibrated with the assistance of the Bureau of Standards prior to the start of the study. All staff members measuring weights were observed by the author to ensure that the procedure of weight estimation was uniform and the use of the scales was accurate.

The measured weights were later compared to their estimated weights as calculated using the APLS formula, the Luscombe and Owens formula and a “best fit” formula derived (then simplified) from linear regression analysis of the measured weights in this sample.

Discussions with expert colleagues in the Department of Pharmacology at the University of the West Indies, St Augustine, were held to determine the percentage divergence that would be clinically significant between estimated and calculated weight. It was decided that a 10% divergence would produce clinically significant differences in patient management, particularly with regard to the potential toxicity of intravenous infusions of drugs with a low therapeutic index such as aminophylline, digoxin and dopamine.

Accuracy of weight estimation methods was compared using three different methods: bias (mean differences between methods compared) and precision (95% limits of agreement) were measured using the Bland-Altman method; in addition, the mean percentage differences between each estimated weight (APLS, Luscome and Ownes and the derived formula) and actual measured weights were compared. Finally, the proportions of patients whose estimated weights fell within 10% of the measured weight for each formula was calculated.

Sample size was estimated using power-based sample size calculations. To detect a 10% difference (δ) between the calculated APLS formula, the Luscombe and Owens formula and the measured weight, when the level of significance (α) is < 0.05 and the power of the study is 80%, a sample size of 252 patients per year of age was needed (See Additional file 1).

The accuracy and precision of each method of weight calculation was estimated using Bland-Altman analysis. In addition, Student’s *t*-test was used for estimation of statistical significance of any difference in variation between estimated weight using different formulae and measured weight. Chi squared testing was used to assess differences in the percentage of patients whose estimated weights fell within 10% of measured weight using different formulae.

The data was collected and tabulated using a Microsoft Excel 2007 spreadsheet. Microsoft Excel 2007 was also used to calculate percentage differences and mean values and to create the graphical representation of the data. Epi Info v. 3.5 was used to calculate standard deviations, confidence intervals and to perform linear regression analysis.

## Results

1784 patients met the eligibility criteria for the study. Of these, 45 (2.5%) were excluded due to weight not being documented in the record and 6 (0.3%) were excluded due to illegible entries. 1723 patients were included in the final analysis.

Using linear regression analysis, the best formula to describe weight and age was as follows: Weight = 2.40 × age + 8.25. This formula, however, is impractical for use in the Emergency Department due to difficulty for quick calculation. Another more applicable formula would be: Weight = 2.5 × age + 8. The APLS formula, the Luscombe and Owens formula ([3 × age] +7) and the newly derived formula ([2.5 × age] + 8) were compared. The results are shown in Table 1.

**Table 1:** Comparison of APLS, Luscome and Owens and new derived formula using Bland-Altman method

**Age at last birthday (years)**	**Number of patients**	**APLS formula**		L**uscombe and Owens formula**		**Derived formula**	
		**Bias (estimated – measured weight - kg)**	**95% Limits of Agreement**	**Bias (estimated – measured weight - kg)**	**95% Limits of Agreement**	**Bias (estimated – measured weight - kg)**	**95% Limits of Agreement**
1	439	−0.6	3.1 to −4.4	−0.6	3.1 to −4.4	−0.15	3.2 to −4.6
2	355	−1.2	4.1 to −6.6	−0.2	5.1 to −5.6	−0.2	5.1 to −5.6
3	318	−1.2	4.3 to −6.7	−0.8	6.3 to −4.7	−0.3	5.2 to −5.8
4	306	−2.0	5.3 to −9.4	1.0	8.3 to −6.4	−0.04	7.4 to −7.5
5	305	−2.4	6.6 to −11.4	1.6	10.6 to −7.4	0.3	8.5 to −7.9
All ages	1723	−1.4	5.0 to −7.8	−0.4	6.9 to −6.1	−0.4	6.0 to −6.1

There is no significant difference in accuracy or precision between the three methods.

The APLS formula underestimated weight in all age groups with a mean difference (bias) of −1.4 kg (95% limits of agreement 5.0 to −7.8). This was most pronounced in the 5 year old age group with a mean difference of −2.4 kg (95% limits of agreement 6.6 to −9.4) and least pronounced in the 1 year old age group with a mean difference of −0.6 kg (95% limits of agreement 3.1 to −4.4). The Luscombe and Owens formula was slightly more accurate in predicting weight than the APLS formula overall with a mean difference of −0.4 kg (95% limits of agreement 6.9 to −6.1) (Table 1). The derived formula was the most accurate in predicting weight, with all age groups having an error of less than 10% with an overall underestimation of −0.4 kg (95% limits of agreement 6.9 to −6.1). The Bland-Altman graphs for each estimated weight formula are shown in Additional file 2.

Accuracy was also compared by calculating the percentage differences between the estimated weights from each formula and the measured (actual) weights of the patients. The overall percentage difference between the estimated weights using the APLS formula compared to the actual weights was −5.8% (95% confidence intervals −5.0 to −6.6). This difference was least marked in the 1 year age group and most marked in the 5 year age group. The overall percentage difference between the estimated weights using the Luscombe and Owens formula and the actual weights was +5.0% (95% confidence intervals 4.1 to 5.9). Again, the difference was more marked with the older age groups of children. The derived formula was most accurate, with a percentage variation of −3.1% (95% confidence intervals −3.9 to −2.3).

The precision of the three formulae was also determined by calculating the percentage of patients whose estimated weight was within 10% of the measured weight, for each formula. Using the APLS formula, 45.6% of children were within 10% of actual measured weights, whereas 42.3% of children were within 10% of actual weights using the Luscombe and Owens formula. Using the derived formula, 47.5% of children would have had estimated weights within 10% of their actual weights. After Chi squared testing, these differences were not found to be statistically significant.

## Discussion

The APLS formula to calculate weight in children is a commonly used method, especially for critically ill children in whom it is impractical or unsafe to acquire weight on a scale. During resuscitation of children, weight is used to guide drug dosages, intravenous (IV) fluid boluses, equipment size, defibrillation and cardioversion dosages. As described in the introduction, there are a number of methods used to estimate weight in children [3-9]. However, At the Eric Williams Medical Sciences Complex, the most commonly used method is the APLS formula.

It has been shown by several studies that the original APLS formula underestimates weight, however all of these studies have been performed on non-Caribbean populations [3-7]. In 2007, Luscombe and Owens examined data from over 17000 children and found that the APLS formula was found to have underestimated weight by a mean of 18.8% [3]. Several subsequent studies in Australia and the United Kingdom also demonstrated the tendency for the APLS formula to underestimate weight in children in developed countries [4-6,9]. This included a review of 93827 children over a 5 year period from 2003 to 2008 by Luscombe et al. [10]. In light of this, the most recent edition of the APLS manual recommends the use of the Luscombe and Owens formula in children aged 6 – 12 years old, with retention of the original APLS formula for those aged 1 – 5 years.

In India, however, Varghese et al. examined 500 outpatient children and found the APLS formula to overestimate weight in their population by a mean of 2–3 kg [7]. In addition, a 2010 study of the accuracy of various weight estimation methods in South African children concluded that the APLS formula and Broselow tape were more accurate than the Luscombe and Owens formula over all age groups in this population [11].

The Broselow tape was designed for use in children from 45 cm to 145 cm in length [5]. Although it has been validated in several studies as a reliable tool for estimating weight [5,7,11-13], it is not commonly used in Trinidad. One of the largest studies of the Broselow tape was performed by Lubitz et al. in the United States of America [1]; out of 937 patients it was found that 79% of patients had estimated weights using the Broselow tape which were within 15% of their actual weights. Krieser et al. compared the Broselow tape to the APLS formula and found it to be more accurate, with 61% of patients having Broselow weights within 10% of actual weights compared to 34% of patients having APLS weights within 10% of actual weights [5]. Interestingly, Cattermole et al. found mid-arm circumference to be more accurate and precise than age-based rules for predicting weight in school aged children, and as accurate as the Broselow tape [14].

Krieser et al. [5] showed that parental estimate of children’s weight was a reliable method of weight estimation; 78% of the 410 children studied had an estimated weight within 10% of their actual weight and the mean difference between estimated and measured weight was −0.6 kg [5]. A previous study performed by Harris et al. had demonstrated that out of 100 children from 0–8 years of age, 84 had estimated weights within 15% of actual weights when parental estimate was used [15]. Leffler et al. also demonstrated that parental estimate was within 10% of actual weights in 80% of cases [16] and Goldman et al. demonstrated that parental estimate was within 10% of actual weights in 73% of cases [17]. This method would have to be tested further in a Trinidadian population to determine whether it is as accurate as in other countries.

It is clear from the previously cited studies from India and South Africa that age-based formulae for estimating weight may not be applicable to developing countries, and that studies on these formulae will yield differing results in different settings, given the variation in body habitus between children from developed and developing countries. In light of this, formulae used in the first world for weight estimation should be tested before they are adopted in developing countries, such as Trinidad.

This study showed that the APLS formula did not significantly underestimate weight in the 1–5 year age group compared to other formulae. This is in contrast to the evidence that has been emerging worldwide, where there has been a tendency for the APLS formula to significantly underestimate weight. This may be in keeping with the UNICEF progress for children report, which found a larger proportion of underweight Trinidadian children than in first world countries [18]. This and other evidence suggests that Trinidadian children in this age group weigh less than their first world counterparts [19].

The Luscombe and Owens formula was no more accurate at estimating weight than the APLS formula in our population. The APLS formula was also found to be marginally more precise than the Luscombe and Owens formula, with 45.6% having estimated weight within 10% of measured weight using the APLS formula as opposed to 42.3% using the Luscombe and Owens formula. The new derived formula ([2.5 × age] + 8) was more accurate than either the APLS or Luscombe and Owens formula. However, the overall accuracy and precision of all three formulae were not found to be significantly different.

These findings suggest that the APLS formula is acceptable for use in the 1–5 year old age group. Although the new formula was slightly better than the APLS formula, it is the recommendation of the authors that the APLS formula be retained for weight estimation. This is because the APLS formula is already familiar to medical staff in Trinidad and Tobago, and the ease of recalling a familiar formula would make it a more practical choice. This is also in light of the fact that adopting the new formula would not produce a significant improvement in weight estimation.

The study has several limitations. Firstly, ethnicity, gender and socioeconomic status were not taken into account. While there may have been some variation in weights based on these factors, it was thought unnecessary to analyse these subgroups separately, as it is unlikely that separate formulae for each of these categories would be practical for use in the emergency situation. In addition, the one study of the relationship between ethnicity and weight in children in Trinidad did not show any significant difference [20].

Secondly, the study did not include children aged 6 years or older, so it is not known whether the formula would be applicable to older children. The younger (pre-school) age group was specifically investigated in this study, as these children make up the majority of patients presenting to the Paediatric Emergency Department. In addition, the authors felt that these smaller, younger patients were more likely to suffer ill effects of miscalculation of dosage of medication than the older age group. However, it is well accepted that children’s weights do not bear a linear relation to age, and it would be necessary to perform a similar study on older children (6 – 12 years) to test the accuracy and precision of the various weight estimation formulae on this age group.

Finally, the study was restricted to the island of Trinidad. While the results of this study may be generalisable to the rest of the Caribbean region, the authors intend to do a more extensive study of weight estimation in children across the Caribbean.

## Conclusion

Estimating children’s weight in critical situations is important in order to maximise the effectiveness and safety of resuscitation. This study found the APLS formula to be superior to the Luscombe and Owens formula in estimating weight when compared across all age groups from 1 – 5 years. The most accurate formula was a formula derived from the children’s actual weights (Weight = [2.5 × age] + 8). The APLS formula, however, is easily calculable, familiar and already widely used in Trinidad and Tobago. In spite of the limitations of the study, it would seem that the APLS formula should continue to be used in the 1–5 year age group in Trinidad. A further study is required to determine whether these findings are reproduced in older children in Trinidad. In addition, more work is required to validate these weight formulae in other Caribbean islands which have more ethnically homogenous and less affluent populations.

## Competing interests

The authors have no competing interests to declare.

## Authors’ contributions

KA conceptualized this study, collected the data and performed the statistical analysis, as well as preparing the manuscript. IS participated in the design of the study, assisted in the review of the literature and assisted in the preparation of the manuscript. PN advised on the study design, including statistical analysis, assisted in the formulation of the discussion, and assisted in interpretation of the study results. All authors read and approved the final manuscript.

## Pre-publication history

The pre-publication history for this paper can be accessed here:

http://www.biomedcentral.com/1471-227X/12/9/prepub

## Supplementary Material

Additional file 1 **Bland-Altman plots for different estimated weights.** This additional file contains three (3) graphs showing the Bland-Altman plots for each of the different methods of weight estimation against measured (actual) weight. Click here for file

Additional file 2 **Bland-Altman plots for different estimated weights.** This additional file contains three (3) graphs showing the Bland-Altman plots for each of the different methods of weight estimation against measured (actual) weight. Click here for file
