# Supplementary material for: Is the APLS formula used to calculate weight-for-age applicable to a Trinidadian population?
Source: BMC Emerg Med. 2012 Aug 2;12:9. doi: 10.1186/1471-227X-12-9 (PMC3506443; doi:10.1186/1471-227X-12-9)
Supplement: Additional file 1 — Bland-Altman plots for different estimated weights. This additional file contains three (3) graphs showing the Bland-Altman plots for each of the different methods of weight estimation against measured (actual) weight. [file 1471-227X-12-9-S1.pdf]

Additional File 1: Sample size calculation for study.

Sample size was estimated using power-based sample size calculations. To detect a 10% difference (δ) between the calculated APLS formula, the Luscombe and Owens formula and the measured weight , when α is < 0.05 and power is 80% , a sample size of 252 patients per year of age was needed.

Previous studies suggest that, when measuring weight differences as a percentage of actual weight of subjects, the standard deviation for the sample would fall between 20% and 40%. Therefore, in the formula below, s (the standard deviation of the sample) was estimated at 30%.

The formula used for estimating sample size was:

$$n= f(\alpha,\beta).2s^2/(\delta)^2$$

Where:

- α= is the significance level
- 1 – β= is the power of your test.
- f(α,β) is a calculated function of α and β.
- s is the standard deviation (estimated from previous studies

	β		
α	0.1	0.2	0.5
0.05	10.5	7.9	3.8
0.01	14.9	11.7	6.6

Table: calculated values of f(α , β) for different set values of α and β.

References:

1. Statistics: An Introduction to Sample Size Calculations Rosie Cornish. 2006. Mathematics Learning Support Centre
(accessed at http://mlsc.lboro.ac.uk/resources/statistics/Samplesize.pdf)
