# Supplementary material for: Is the APLS formula used to calculate weight-for-age applicable to a Trinidadian population?
Source: BMC Emerg Med. 2012 Aug 2;12:9. doi: 10.1186/1471-227X-12-9 (PMC3506443; doi:10.1186/1471-227X-12-9)
Supplement: Additional file 2 — Bland-Altman plots for different estimated weights. This additional file contains three (3) graphs showing the Bland-Altman plots for each of the different methods of weight estimation against measured (actual) weight. [file 1471-227X-12-9-S2.pdf]

Additional File 2: Bland-Altman plots for different estimated weights

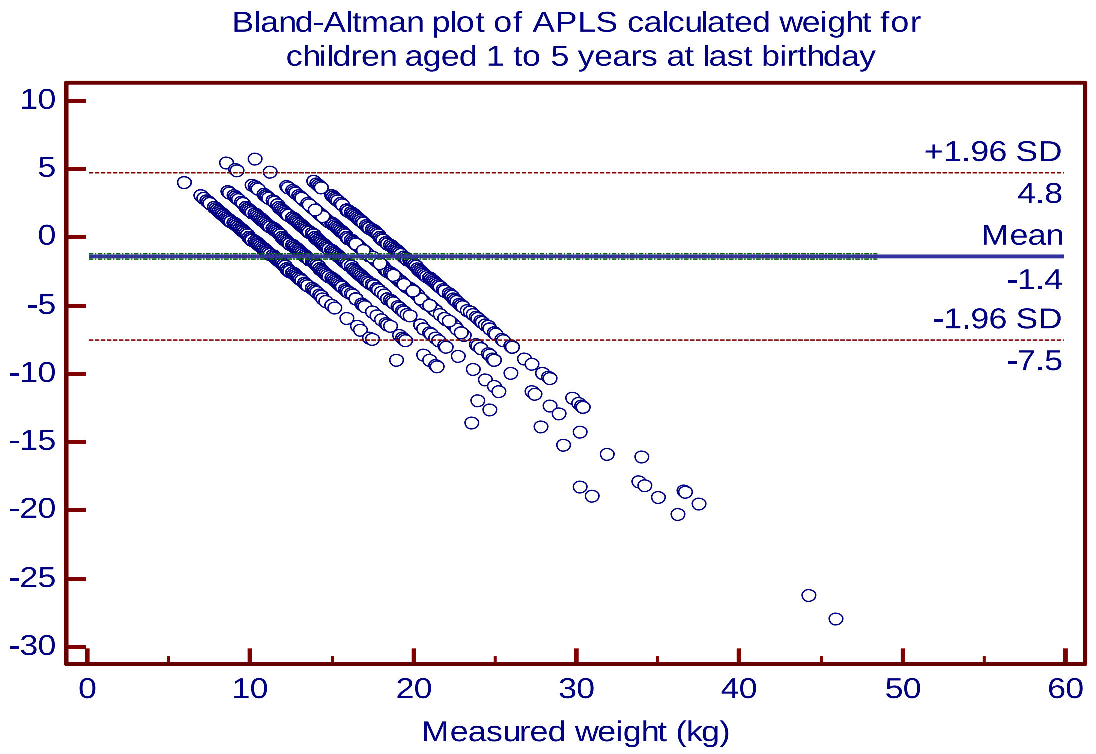

Figure A: Bland-Altman plot of APLS estimated weight versus measured weight.

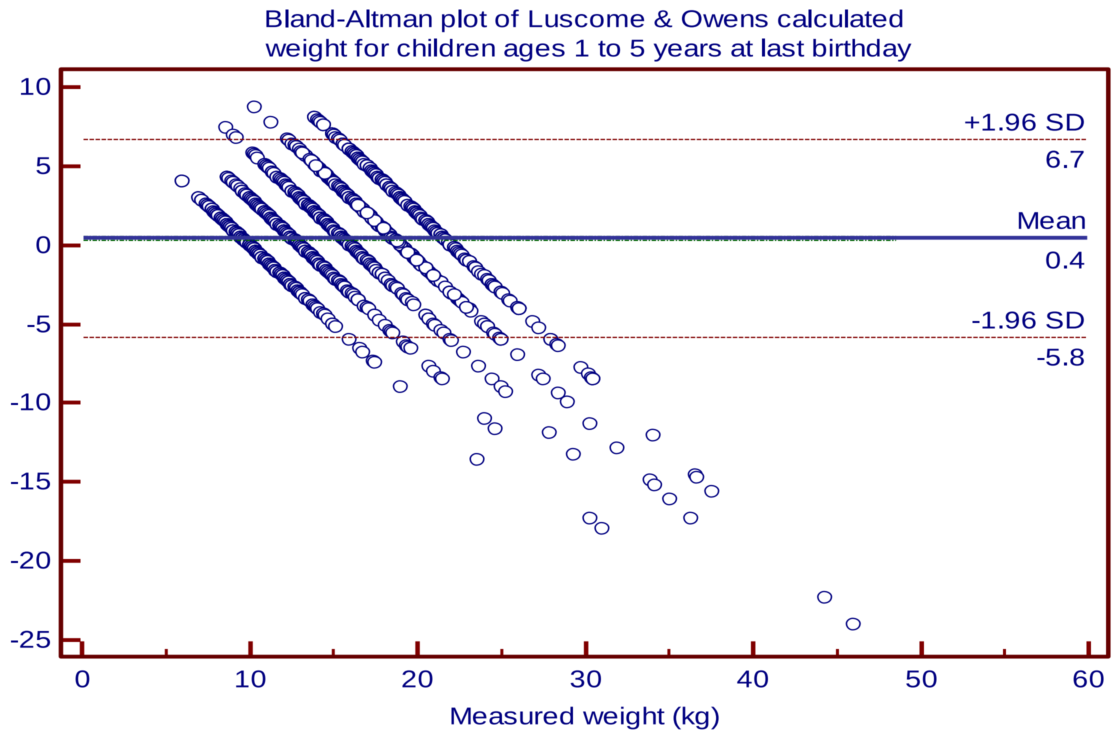

Figure B: Bland-Altman plot of Luscome and Owens estimated weight vs measured weight

–

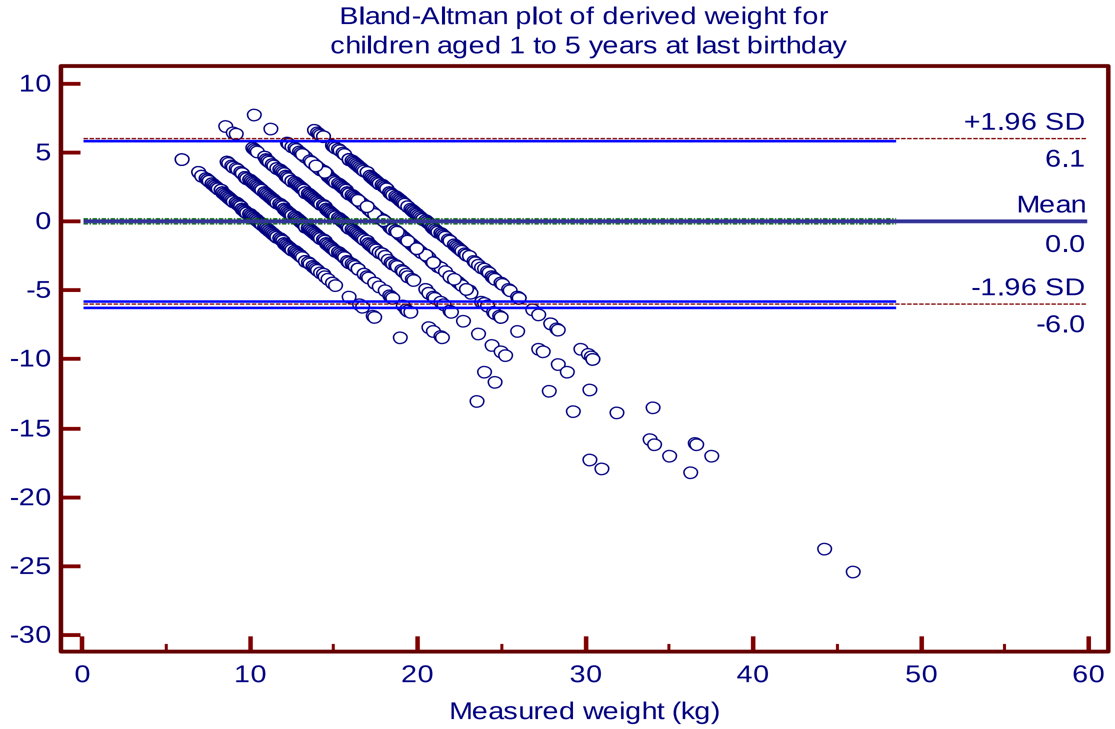

Figure C: Bland-Altman plot of estimated weight (using derived formula) vs measured weight
